# Effect of Two Different Ageing Exposures on the Colour Stability of Transparent Polyurethane Finishing

**DOI:** 10.3390/polym15153313

**Published:** 2023-08-05

**Authors:** Gabriela Slabejová, Zuzana Vidholdová, Mária Šmidriaková

**Affiliations:** 1 Faculty of Wood Sciences and Technology, Department of Furniture and Wood Products, Technical University in Zvolen, 960 01 Zvolen, Slovakia; slabejova@tuzvo.sk (G.S.); smidriakova@tuzvo.sk (M.Š.); 2 Faculty of Wood Sciences and Technology, Department of Wood Technology, Technical University in Zvolen, Masaryka 24, 960 01 Zvolen, Slovakia

**Keywords:** alder, beech, birch, colour, dark, hydrothermally treated wood, light, maple, polyurethane finishing

## Abstract

This paper deals with the influence of dark and light exposure on the colour change of a transparent two-component polyurethane surface finish. The surface finish with polyacrylic and aldehyde resin was applied to the surfaces of untreated and hydrothermally treated European beech, alder, Norway maple, and Paper birch wood. The test specimens were deposited indoors for 60 days. The colour values (lightness L*, redness + a*, yellowness + b*, chroma C*, hue angle h°) were expressed in the CIELAB system. The results showed that the colour difference of the finish surfaces of all hydrothermally treated wood species was 27–50% lower after the dark than when exposed to light. In the case of finished untreated wood, the colour difference was 51–73% lower after the dark than light exposure. Only the finished untreated and hydrothermally treated Norway maple wood surfaces showed similar and significant changes after both dark and light exposure. The lower value of the colour difference of the finished hydrothermally treated wood was due to the fact that steaming wood with saturated water steam has a positive effect on the overall colour stability of the finish and partial resistance to the initiation of photolytic reactions caused by light.

## 1. Introduction 

The first polyurethanes were produced more than eighty years ago. The main raw materials in ain polyurethane production are diol and isocyanate, which react to form urethane linkage [1,2]. Polyurethanes are used in medical science, automobiles, coatings, adhesives, sealants, paints, textiles, etc. For polyurethane coating materials, the basic components are compounds containing reactive isocyanate groups, and the additional components are substances that have free hydroxyl groups, e.g., polyester, alkyds and polyacrylates [3]. Two-component polyurethane coatings are supplied to the market as a solution of polyhydroxy esters and an additional solution of polyisocyanates (the hardener). Polyurethane coating materials are characterised by good resistance to water, chemicals, mechanical stress, and higher temperatures. Currently, conventional petroleum-based polyurethane coatings have been replaced by their green counterparts, i.e., bio-based polyurethane coatings [4]. The second group, in addition to solvent polyurethane coating materials, is made up of water-based polyurethanes. Polyurethane aqueous dispersions are becoming increasingly important in the coatings industry [5,6,7]. In the production of polyurethane substances, sustainability must be taken into account, as well as the possibility of improving the properties of coating materials by various modifications [8,9,10,11,12,13,14]. Polyurethane coating materials are used to protect various materials based on metals or wood. They are produced as pigmented or transparent. In the interior for wood finishing, we prefer transparent coating materials that preserve the appearance of wood and protect it from wear and tear [15,16,17]. However, coatings are generally not able to provide sufficient protection because UV light can penetrate through the film layer and cause degradation of the wood and even the coating itself [18]. Transparent or clear surface finishing is designed to enhance the stability of the wood surface and maintain the natural aspects of the wood, such as the colour, grain, and texture, for a long time. Transparent finishing films perform badly on wood surfaces during interior or exterior exposure. In fact, these types of coatings cannot absorb UV light, and attend the wood surface [19]. This phenomenon leads to the photodegradation of the wood substrate. The surface cellulose layer has the potential to stabilise the wood surface against discolouration. However, it can be affected by UV light. Under UV light, a decrease in lignin content and, at the same time, an increase in the crystalline cellulose content on the surface [20]. A visible colour change in wood is the first sign of its chemical modification when exposed to light, even in diffuse indoor light conditions. Changes in the colour of the wood surface after applying a transparent coating material are the result of an interaction of the colour of the coating film with the colour of the wood surface [21]. But we know from practice that not always the entire surface of a wood product (e.g., floor, shelf, table) is exposed to light. Many scientific works deal with the change in wood colour [22,23,24,25,26,27] or the change of surface finishes on wood in the interior due to the influence of light [17,28,29,30] or after accelerated weathering [9,13,31,32,33,34,35,36]. However, the effect of visible light from short waves (violet-blue) and the thermic effect of the infrared components of natural light passing through window glass [36,37,38] have to be considered as indoor ageing factors [25,39,40]. Fewer works pay attention to wood ageing and to surface finishes that are not directly exposed to light [25,28].

The aim of the presented work was to evaluate the effect of dark and light exposure on the colour change of the transparent two-component surface treatment on untreated (native) and hydrothermally modified wood species with the aim of adding new useful data in this area.

## 2. Materials and Methods

### 2.1. Materials

In this study, a transparent two-component polyurethane surface finish based on polyacrylic and aldehyde resin. Its commercial name is PUR SL-212-Schichtlack/30. It is recommended for interior use on solid wood, veneers, tables and worktops, and kitchen and bathroom furniture. It is highly scratch resistant and fully built. It was applied by spraying in two coats with a spread rate of 80–120 mL·m^−2^. The density of the coating material is 0.94 g·cm^−3^. The thickness of the dry film was 36 ± 2 μm. The samples of untreated and hydrothermally treated (HTT) wood of four wood species (Table 1) were prepared from six-month air-conditioned and sanded boards (sanded first transversely and then in the longitudinal direction, the last sand grit P 180). 

The samples containing mature wood had three to eight growth rings per cm, they were free from defects, and the growth ring orientation to the tested surface was 5° to 45°. After application, the samples were stored in a dark room at 23 °C and 50% relative humidity for 14 days to ensure film formation, sufficient hardening, and solvent evaporation.

### 2.2. Ageing Exposures

The samples exposed to ageing exposures were carried out for 60 days in the summer months. The coated and uncoated samples were stored in a room in the interior: (1) in dark condition (packed in aluminium foil) and (2) in light condition (exposed behind a glass window—thermal-insulation double glazing with U-factor 1.1 W·m^−2^·K^−1^ with west direction). The interior temperature ranged from 20 to 25 °C, and relative humidity varied from 50% to 55%. The daily average of the total solar power density was between 336 and 535 W·m^−2^ in Zvolen, Slovakia (Table 2). The geographical data for Zvolen are longitude 19°07′03″ East, latitude 48°34′15″ North, and an altitude of 283 m. 

### 2.3. Evaluation of Colour Change

**Figure 1:**
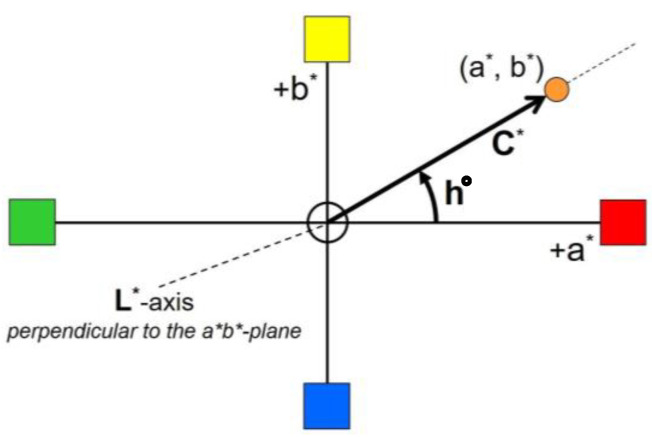
The CIELAB colour system.

The colour changes of the sample surfaces were visualised and quantified by using the CIELAB colour space. The 3-dimensional colour space is built up from three axes. The L*-axis gives the lightness: a white surface has an L* value of 100, and the L* value of black surfaces is 0. The so-called achromatic colours, the shades of grey, are on the L*-axis. Chromatic (‘real’) colours are described by using the two axes in the horizontal plane. The a*-axis is the green-red axis, and the b*-axis goes from blue (−b*) to yellow (+b*). Hue is the colour tone or name of a colour. Chroma is the amount of saturation of a colour. Colours of high chroma are said to be clear, bright, or brilliant. Dull (pastel) colours have a low chroma. Hue and chroma can be visualised and quantified by using the a*b*-plane of the CIELAB colour space (Figure 1). 

The colour parameters of the tested samples were measured using a Colour Reader CR-10 (Konica Minolta, Osaka, Japan) after surface finishing and after exposition in the dark or in the light. The device was set to an observation angle of 10°, with d/8 geometry, and a D65 light source. The colour values (lightness L*, redness + a*, yellowness + b*, chroma C*, hue angle h°) were measured at the 10 given positions on each of the tested samples tested and expressed in the CIELAB system [41]. 

The colour changes of the sample surfaces were measured after 60 days. Total colour difference $∆E_{ab}^{*}$ was subsequently calculated as the Euclidean distance between the points that represent them in the space using the following equation [41,42]

(1) $$∆E_{ab}^{*}=\sqrt{∆L^{*2}+∆a^{*2}+∆b^{*2}}$$

where: ΔL*, Δa*, Δb* are the differences in individual axes (the difference between the value measured after 60 days of exposure in dark and light and before exposure). The magnitude of $∆E_{ab}^{*}$ can be classified according to the classification reported in Table 3.

To demonstrate the colour change of the coated wood surfaces (before and after the exposure), the scanner Colour Laser Jet Pro MFP M477fdw was used.

### 2.4. Statistical Evaluation

MS Excel 2013 and statistical software STATISTICA 12 was used to analyse and present the collected data on colour parameters. Descriptive statistics deal with basic statistical characteristics—average and standard deviation—and an analysis of variance (ANOVA) followed by the Duncan test. 

## 3. Results and Discussion

**Figure 2:**
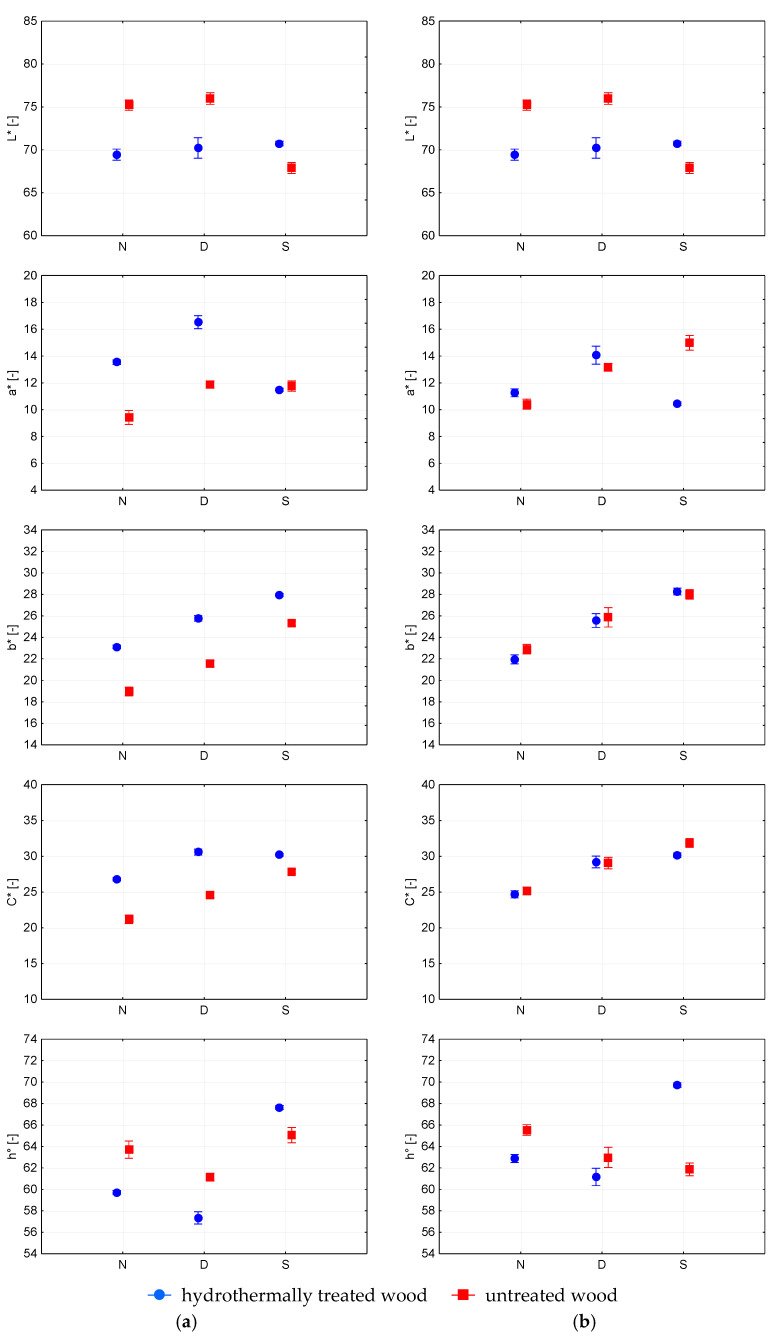
Colour coordinates L*, a*, b*, C* and h° for untreated and hydrothermally treated: (**a**) European beech (*Fagus sylvatica* L.) and (**b**) alder (*Alnus glutinosa* (L.) Gaertn.) with polyurethane surface finish before finish (N) and after ageing in dark (D) and light (S) condition.

The surface finish on HTT beech wood did not change lightness in the dark, whereas in the light, it lightened highly significantly (>99.9%). This was also confirmed by the Duncan test. Additionally, the change in the lightness of the surface finish on untreated beech wood showed a low significance (>95%) in the dark (Table 4, Table 5 and Table 6, Figure 2a). 

**Figure 3:**
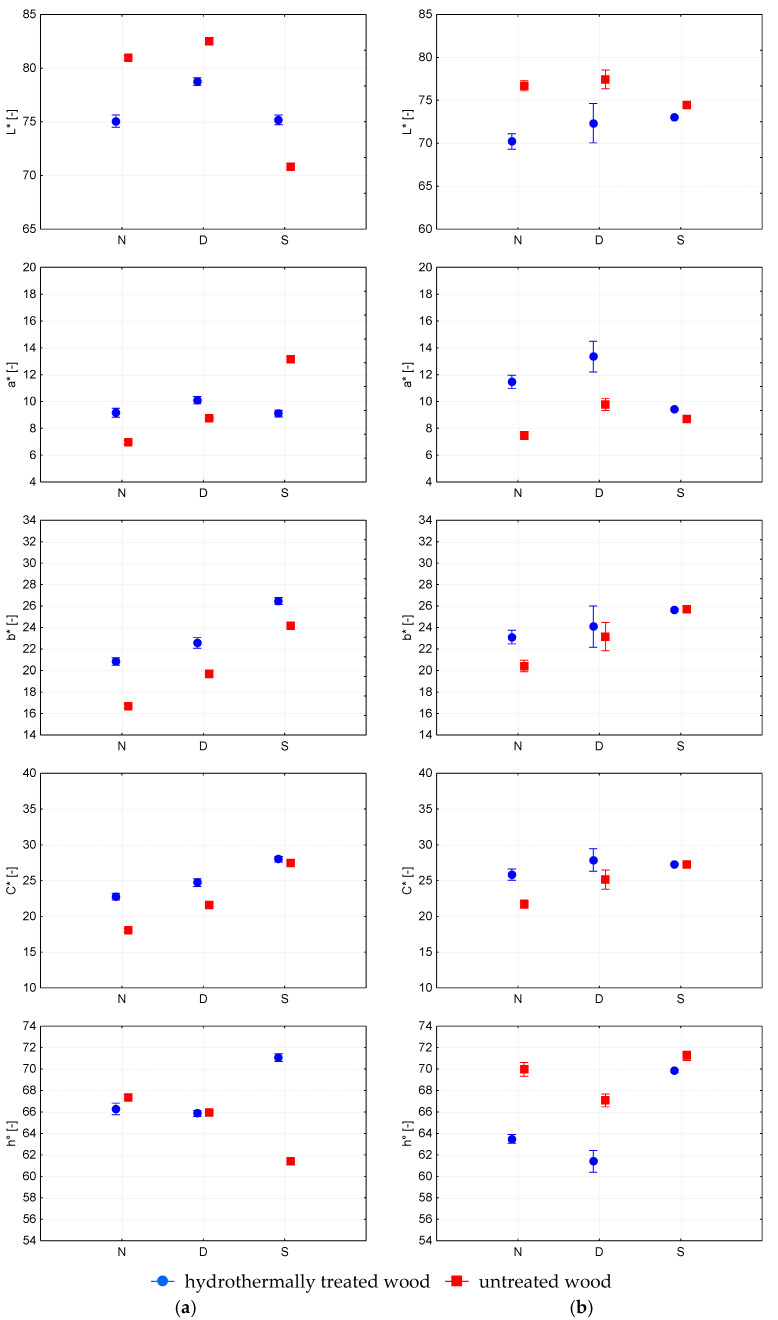
Colour coordinates L*, a*, b*, C* and h° for untreated and hydrothermally treated: (**a**) Norway maple (*Acer pseudoplatanus* L.); and (**b**) Paper birch wood (*Betula papyrifera* Marsh) with polyurethane surface finish before the finish (N) and after ageing in dark (D) and light (S) condition.

The change in the lightness of the surface finish on HTT alder wood was insignificant in the dark (<95%), whereas the surface darkened highly significantly in the light (>99.9%). The surface finish on the untreated alder wood lightened low significantly in the dark (>95%) and significantly darkened in the light (>99%) (Figure 2b). This was also confirmed by the Duncan test (Table 4). Figure 3a shows that the change in the lightness of the surface finishes on the HTT maple wood was statistically highly significant (>99.9%) in the dark. The change in the lightness of the surface finish on untreated maple wood: the surface finish lightened up highly significantly (>99.9%) in the dark and became darker in the light. Figure 3b shows that the change in the lightness of the surface finishes on HTT birch wood was insignificant in the dark (<95%), and the surface highly significantly lightened in the light (>99.9%). The surface finish on the untreated birch wood did not change the lightness in the dark (<95%), and the surface darkened highly significantly in the light (>99.9%).

In the dark, the colour differences on the tested surface finish were graded as “Colour difference visible with medium-quality screen” or “Colour difference visible with high-quality screen”. In light, the surface finish reached a “High colour difference” on European beech, Alder and Norway maple untreated maple wood (Table 5 and Table 6). The authors [5] state that the colour differences of polyurethane surface finish on Oriental beech, before and after 250 h of accelerated weathering, was 18.30, which was a “Different colour”. The paper [27] reports “Colour difference visible with medium quality screen” for oil surface finishes on European maple wood after accelerated simulation in the dark. The tested surface finish on untreated Paper birch wood showed “Colour difference visible with medium quality screen”. Different values of colour differences in the surface finish on different types of wood are caused by the interaction of the colour change colour of the wood surface and the colour change of the coating film itself. The different behaviour of the wood species studied may be related to the differences in their chemical composition, especially lignin and extractives content. Wood extractives are an important factor affecting wood colour, while also playing an important role in the wood photodegradation process [23,26,27,28]. Different sizes of colour differences of the surface finish in the dark from those in the light point to the significant influence of light. Studies [24,28] are highlighting the role of light as the main factor in indoor natural ageing, while also pointing towards degradation of lignin as a main photo-chemically induced process. 

The hydrothermal modification of the wood reduced the colour differences of the surfaces with the surface finish exposed to light. Colour differences showed “Colour difference visible on medium quality screen”. Only in the case of Norway maple HTT wood the colour difference achieved a “High colour difference (Table 4). The authors in [27] claim that the surface colour of untreated maple wood changes more markedly than the surface colour of steamed maple wood due to UV radiation. The size of the colour difference $∆E_{ab}^{*}$ of the surface finish on untreated wood, and HTT wood was different in the dark from that in the light. The authors [28] claim that some small colour changes occurred in the surface finish, which is protected from the direct action of light; these changes were due to thermal-induced ageing. Of course, the differences of the individual coordinates ΔL*, Δa*, Δb*, ΔC*, Δh° were also different in the dark from those in the light. The coordinate a* of the surface finish on both the untreated and HTT surfaces in the dark moved toward red colours (Figure 2 and Figure 3). When exposed to the light, the surface finish on untreated wood shifted predominantly towards red colours; on the HTT wood, towards green colours (Figure 2 and Figure 3). The b* coordinate of the surface finish on both untreated wood and HTT wood shifted predominantly in the direction of yellow colours in the dark as well as in the light. These statements are also confirmed by work [27]. The shift of the b* coordinate was significantly smaller in the dark than in the light. Yellowing was mostly associated with lignin degradation [24,28], and these results suggest that UV light penetrated through the coating layer affecting lignin at the interface wood/coating, which is in accordance with previously reported research [17]. The study [29] states that the change in Δb* was most strongly influenced by the number of polyurethane varnish layers. Similar changes to those on the b* coordinate were also recorded for the C* coordinate. Figure 2 and Figure 3 show that the angle h° of the surface finish on the HTT wood surfaces for the four wood species has the same direction in the dark. The angle increases in the light and decreases in the dark. On the surface finish on untreated wood in the dark, the h° angle decreases for all four species of wood. But in the light, the angle increases on European beech and Paper birch, and it decreases on alder and Norway maple.

The interaction between the colour of the coating film and the colour of the wood surface causes changes in the colour of the wood surface after the application of the transparent coating material [21]. The fact that hydrothermal treatment of wood with saturated water steam has a good impact on the overall colour stability of the finish and partial resistance to the beginning of photolytic processes generated by light [27,44,45] is the reason why the colour difference of the completed hydrothermally treated wood was less noticeable. The authors of [27] used FTIR analysis of chemical changes in HTT and untreated maple wood. Using FTIR, they observed the formation of new carbonyl C=O groups. These could come not only from the main constituents of wood (lignin, cellulose, and hemicelluloses), but also from extractives. They recorded the most significant increase in the intensity of the C=O group bands in the spectrum of the sunlight ageing untreated wood than treated wood. The authors [44] stated that the lightfastness of beech wood was also greatly affected by lignin. The main change in the ATR-FTIR spectra was the decrease in lignin absorption bands in parallel with the increase in unconjugated carbonyl absorption after the ageing of HTT and untreated wood with daylight components. Photodegradation of wood was also associated with a reduction in the ratio of lignin to carbohydrates while at the same time increasing the ratio of unconjugated carbonyl to carbohydrates, which may affect the partial resistance of HTT beech wood to daylight. The authors [45] suggested that the lower value of the colour difference of HTT birch wood points to the fact that hydrothermal treatment of wood with saturated water steam has a positive effect on colour stability and partial resistance to the initiation of photolytic reactions caused by sunlight. 

Photo-oxidative degradation and yellowing or discolouration in polyurethane coatings upon UV irradiation is a general phenomenon. In our previous study [30], discolouration of a finishing system based on polyacrylic, and aldehyde resin resulted mainly from the photo yellowing of underlying untreated and HTT-treated wood due to sunlight and increased with irradiation time. Furthermore, the results of the study [46] showed that an accelerated ageing process with simulation of indoor conditions induced significant discolouration of surface-treated wood, both coated with solvent-based polyurethane and water-based lacquers. The spruce wood surfaces coated with solvent-based polyurethane lacquers were less stable than the oak surfaces treated in the same ways. There were also confirmed significant impacts for wood species, lacquer/coating system type, lacquer modification, and wood surface pre-treatment with pigment and stain mordants on colour change during accelerated ageing. The better colour resistance of transparent surface-finished wood confirms the already recognised fact that darker wood surfaces are more stable [28,29,30,46]. In addition, it may be affected by the type and amount of topcoat applied [47,48].

## 4. Conclusions

Polyurethane coating material applied to untreated wood and hydrothermally treated wood protects the surface and enhances it aesthetically. At the same time, the coating material creates a coating that changes the colour of the wood surface. If exposed to the dark or to the light, the surface finish changes the colour of the surface.

If evaluated from a practical point of view, products finished with the surface finish will change the colour of the surface significantly less in the dark than in light exposure. Wood species characterised by brown-pink shades (European beech, alder), as well as light wood with yellow-brown shades (Norway maple and Paper birch) with surface finish, became lighter in the dark, while, in the light, they became darker. If exposed to the light for 60 days, the colour change reached “High colour difference” for European beech, alder, and Norway maple. In the dark, the colour difference of the surface finish on HTT wood was comparable to the colour difference of the surface finish on untreated wood.

Thermal treatment of wood did not eliminate the colour difference when the surface was exposed to dark, but it ensured the elimination of the colour difference when the surface was exposed to light. The surface finish on HTT wood showed a colour difference of 27–50% greater in the light than in the dark. The surface finish on the untreated wood showed a colour difference of 51–73% greater when exposed to light in comparison with the dark. In light of this, the surface finish on HTT wood showed a colour difference of 27–56% smaller than the surface finish on untreated wood. In the dark, the surface finish on HTT wood and also on untreated wood became lighter. If exposed to light, the surface finishes on the HTT wood mainly lightened, and on the untreated wood, it darkened.

## Figures and Tables

**Table 1:** Wood species, wood treatment, and ageing exposure were used in the experiment.

Wood Species	Wood Treatment	Ageing Exposure
Alder (*Alnus glutinosa* (L.) Gaertn)	Untreated Hydrothermally treated (HTT) with saturated water steam at the temperature of 135 ± 2.5 °C for 6 h	Dark Light
European beech (*Fagus sylvatica* L.)		
Paper birch (*Betula papyrifera* Marsh)		
Norway maple (*Acer pseudoplatanus* L.)		

**Table 2:** Weather data acquired during ageing progress.

	Radiation [kWh·m^−2^]		Temperature [°C]	
	Average	Standard Deviation	Average	Standard Deviation
July	5.215	1.383	19.6	2.4
August	4.496	1.081	20.5	1.6
September	3.237	1.204	15.5	2.8

**Table 3:** Colorimetric evaluation [43].

$\mathrm{The}\;\mathrm{Scale}\;\mathrm{of}\;\mathrm{Total}\;\mathrm{Colour}\;\mathrm{Difference}\;∆E_{ab}^{*}$	Description
$0.2\;>\;∆E_{ab}^{*}$	Not visible difference
$0.2\;<\;∆E_{ab}^{*}$ < 2	Small difference
$2\;<\;∆E_{ab}^{*}$ < 3	Colour difference visible with high-quality screen
$3\;<\;∆E_{ab}^{*}$ < 6	Colour difference visible with medium-quality screen
$6\;<\;∆E_{ab}^{*}$ < 12	High colour difference
$∆E_{ab}^{*}$ > 12	Different colours

**Table 4:** Duncan test for four species of wood.

Colour Coordinates	Exposition	Wood Species							
		European Beech		Alder		Norway Maple		Paper Birch	
		Untreated	HTT	Untreated	HTT	Untreated	HTT	Untreated	HTT
L*	Dark	◯	–	◯	–	◯◯◯	◯◯◯	–	–
	Light	◯◯◯	◯◯◯	◯◯◯	◯◯	◯◯◯	–	◯◯◯	◯◯◯
a*	Dark	◯◯◯	◯◯◯	◯◯◯	◯◯◯	◯◯◯	◯◯	◯◯◯	◯◯◯
	Light	◯◯◯	◯◯◯	◯◯◯	◯◯◯	◯◯◯	–	◯◯◯	◯◯◯
b*	Dark	◯◯◯	◯◯◯	◯◯◯	◯◯◯	◯◯◯	◯◯◯	◯◯◯	–
	Light	◯◯◯	◯◯◯	◯◯◯	◯◯◯	◯◯◯	◯◯◯	◯◯◯	◯◯◯
C*	Dark	◯◯◯	◯◯◯	◯◯◯	◯◯◯	◯◯◯	◯◯◯	◯◯◯	◯
	Light	◯◯◯	◯◯◯	◯◯◯	◯◯◯	◯◯◯	◯◯◯	◯◯◯	◯◯
h°	Dark	◯◯◯	◯◯◯	◯◯◯	◯◯	◯◯◯	–	◯◯◯	◯◯◯
	Light	◯◯	◯◯	◯◯◯	◯◯◯	◯◯◯	◯◯◯	◯	◯◯◯

Note: HTT = Hydrothermally treated wood. Indexes of the Duncan test characterising the significance level of colour coordinates in relation to the state before exposure: ◯◯◯ high significant decrease > 99.9%, ◯◯ significant decrease > 99%, ◯ low significant decrease > 95%, insignificant decrease < 95%.

**Table 5:** Scans and the colour difference $∆E_{ab}^{*}$ of the surface finish on untreated wood.

Exposition	European Beech	Alder	Norway Maple	Paper Birch
Before	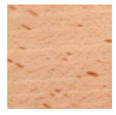	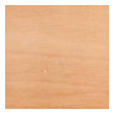	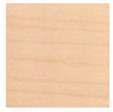	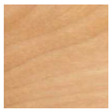
Dark	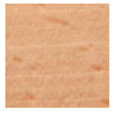	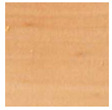	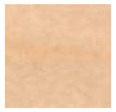	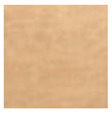
	$∆E_{ab}^{*}$ = 2.7	$∆E_{ab}^{*}$ = 3.1	$∆E_{ab}^{*}$ = 3.4	$∆E_{ab}^{*}$ = 2.8
Light	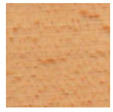	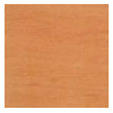	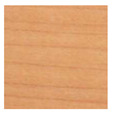	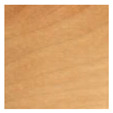
	$∆E_{ab}^{*}$ = 7.7	$∆E_{ab}^{*}$ = 8.9	$∆E_{ab}^{*}$ = 12.6	$∆E_{ab}^{*}$ = 5.7

**Table 6:** Scans and the colour difference $∆E_{ab}^{*}$ of the surface finish on hydrothermally treated woods.

Exposition	European Beech	Alder	Norway Maple	Paper Birch
Before	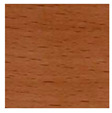	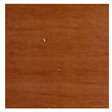	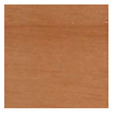	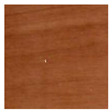
Dark	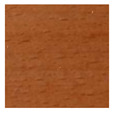	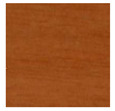	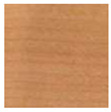	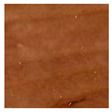
	$∆E_{ab}^{*}\;$= 2.7	$∆E_{ab}^{*}\;$= 3.7	$∆E_{ab}^{*}\;$= 4.1	$∆E_{ab}^{*}\;$= 2.3
Light	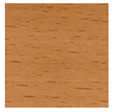	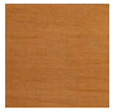	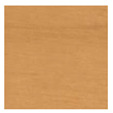	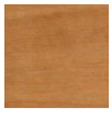
	$∆E_{ab}^{*}\;$= 5.4	$∆E_{ab}^{*}$= 6.5	$∆E_{ab}^{*}$= 5.6	$∆E_{ab}^{*}\;$= 3.8

## Data Availability

The data that support the findings of this study are available on request from the corresponding author.

## References

[1] Delebecq E., Pascault J., Boutevin B., Ganachaud F.F. (2013). On the versatility of urethane/urea bonds: Reversibility, blocked. Chem. Rev..

[2] Wilkes L. (1984). Structure property studies of polyester-and polyether-based mdi-bd segmented polyurethanes: Effect of one- vs. two-stage polymerization conditions. J. Appl. Polym. Sci..

[3] Ionescu M. (2005). Chemistry and Technology of Polyols for Polyurethanes.

[4] Paraskar P.M., Prabhudesai M.S., Hatkar V.M., Kulkarni R.D. (2021). Vegetable oil based polyurethane coatings—A sustainable approach: A review. Prog. Org. Coat..

[5] Scrinzi E., Rossi S., Deflorian F., Zanella C. (2011). Evaluation of aesthetic durability of waterborne polyurethane coatings applied on wood for interior applications. Prog. Org. Coat..

[6] Delpech M.C., Coutinho F.M. (2000). Waterborne anionic polyurethanes and poly (urethane-urea) s: Influence of the chain extender on mechanical and adhesive properties. Polym. Test..

[7] Chattopadhyay D.K., Raju K.V.S.N. (2007). Structural engineering of polyurethane coatings for high performance applications. Prog. Polym. Sci..

[8] Osterhold M., Wagner G. (2002). Methods for characterizing the mar resistance. Prog. Org. Coat..

[9] Rafiee Z., Keshavarz V. (2015). Synthesis and characterization of polyurethane/microcrystalline cellulose bionanocomposites. Prog. Org. Coat..

[10] Calovi M., Rossi S. (2023). From wood waste to wood protection: New application of black bio renewable water-based dispersions as pigment for bio-based wood paint. Prog. Org. Coat..

[11] Das A., Mahanwar P. (2020). A brief discussion on advances in polyurethane applications. Adv. Ind. Eng. Polym. Res..

[12] Calovi M., Coroneo V., Palanti S., Rossi S. (2023). Colloidal silver as innovative multifunctional pigment: The effect of Ag concentration on the durability and biocidal activity of wood paints. Prog. Org. Coat..

[13] Bergamasco S., Tamantini S., Zikeli F., Vinciguerra V., Scarascia Mugnozza G., Romagnoli M. (2022). Synthesis and characterizations of eco-friendly organosolv lignin-based polyurethane coating films for the coating industry. Polymers.

[14] Peng Y., Wang Y., Chen P., Wang W., Cao J. (2020). Enhancing weathering resistance of wood by using bark extractives as natural photostabilizers in polyurethane-acrylate coating. Prog. Org. Coat..

[15] Yan X., Qian X., Lu R., Miyakoshi T. (2018). Comparison and optimization of reactive dyes and coating performance on Fraxinus mandshurica Veneer. Polymers.

[16] Rosu R., Bodîrlau C., Teaca A., Rosu L., Varganici C.D. (2016). Epoxy and succinic anhydride functionalized soybean oil for wood protection against UV light action. J. Clean. Prod..

[17] Capobianco G., Calienno L., Pelosi C., Scacchi M., Bonifazi G., Agresti G., Picchio R., Santamaria U., Serranti S., Monaco A.L. (2017). Protective behaviour monitoring on wood photo-degradation by spectroscopic techniques coupled with chemometrics. Spectrochim. Acta Part A Mol. Biomol. Spectrosc..

[18] Grüneberger F., Künniger T., Huch A., Zimmermann T., Arnold M. (2015). Nanofibrillated cellulose in wood coatings: Dispersion and stabilization of ZnO as UV absorber. Prog. Org. Coat..

[19] Bulian F., Graystone J.A. (2009). Wood Coatings—Theory and Practice.

[20] Evans P.D., Thay P.D., Schmalz K.J. (1996). Degradation of wood surfaces during natural weathering. Effects on lignin and cellulose and on the adhesion of acrylic latex primers. Wood Sci. Technol..

[21] Slabejová G., Šmidriaková M. (2020). Colour of thermally modified wood finished with transparent coatings. Trieskové A Beztrieskové Obrábanie Dreva (Chip Chipless Woodwork. Process.).

[22] Zahri S., Belloncle C., Charrier F., Pardon P., Quideau S., Charrier B. (2007). UV light impact on ellagitannins and wood surface colour of Europena oak (*Quercus petraea* and *Quercus robur*). Appl. Surf. Sci..

[23] Chang T.C., Chang H.T., Wu C.L., Chang S.T. (2010). Influences of extractives on the photodegradation of wood. Polym. Degrad. Stab..

[24] Pandey K.K. (2005). Study of the effect of photo-irradiation on the surface chemistry of wood. Polym. Degrad. Stab..

[25] Timar M.C., Varodi A.M., Gurău L. (2016). Comparative study of photodegradation of six wood species after short-time UV exposure. Wood Sci. Technol..

[26] Dudiak M., Dzurenda L. (2021). Changes in the physical and chemical properties of alder wood in the process of thermal treatment with saturated water steam. Coatings.

[27] Dzurenda L., Dudiak M., Výbohová E. (2022). Influence of UV radiation on the color change of the surface of steamed maple wood with saturated water steam. Polymers.

[28] Liu X., Timar M.C., Varodi A.M., Nedelcu R., Torcătoru M.J. (2022). Colour and surface chemistry changes of wood surfaces coated with two types of waxes after seven years exposure to natural light in indoor conditions. Coatings.

[29] Bekhta P., Krystofiak T., Lis B., Bekhta N. (2022). The impact of sanding and thermal compression of wood, varnish type and artificial aging in indoor conditions on the varnished surface color. Forests.

[30] Vidholdová Z., Slabejová G. (2021). Colour stabilisation of surface of four thermally modified woods with saturated water vapour by finishes. Polymers.

[31] Altay Ç., Toker H., Baysal E., Babahan İ. (2022). Some surface characteristics of oriental beech wood impregnated with some fire-retardants and coated with polyurea/polyurethane hybrid and epoxy resins. Maderas Cienc. Tecnol..

[32] Gündüz A., Baysal E., Türkoğlu T., Küçüktüvek M., Altay Ç., Peker H., Toker H. (2019). Accelerated weatherıng performance of Scots Pine preimpregnated with copper based chemicals before varnish coating. Part II: Coated with water based varnish. Wood Res..

[33] Srinivas K., Pandey K.K. (2017). Enhancing photostability of wood coatings using titanium dioxide nanoparticles. Wood Is Good: Current Trends and Future Prospects in Wood Utilization.

[34] George B., Suttie E., Merlin A., Deglise X. (2005). Photodegradation and photostabilisation of wood–the state of the art. Polym. Degrad. Stab..

[35] Kúdela J., Sikora A., Svocák J. (2020). Colour stability of spruce wood surface coated with a polyurethane lacquer without and with a UV absorber admixture. XIII. Konference Pigmenty a Pojiva: Sbornik/Conference Preceedings.

[36] Reinprecht L., Tiňo R., Šomšák M. (2020). The impact of fungicides, plasma, uv-additives and weathering on the adhesion strength of acrylic and alkyd coatings to the norway spruce wood. Coatings.

[37] Almutawa F., Vandal R., Wang S.Q., Lim H.W. (2013). Current status of photoprotection by window glass, automobile glass, window films, and sunglasses. Photodermatol. Photoimmunol. Photomed..

[38] Serrano M.A., Moreno J.C. (2020). Spectral transmission of solar radiation by plastic and glass materials. J. Photochem. Photobiol. B Biol..

[39] Živković V., Arnold M., Radmanović K., Richter K., Turkulin H. (2014). Spectral sensitivity in the photodegradation of fir wood (*Abies alba* Mill.) surfaces: Colour changes in natural weathering. Wood Sci. Technol..

[40] Persze L., Tolvaj L. (2012). Photodegradation of wood at elevated temperature: Colour change. J. Photochem. Photobiol. B Biol..

[41] (1984). Paints and Varnishes—Colorimetry—Part 3: Calculation of Colour Differences.

[42] (2016). Standard Practice for Calculation of Color Tolerances and Color Differences from Instrumentally Measured color Coordinates.

[43] Cividini R., Travan L., Allegretti O. White beech: A tricky problem in drying process. Proceedings of the International Scientific Conference on Hardwood Processing.

[44] Dudiak M., Dzurenda L., Kučerová V. (2022). Effect of sunlight on the change in color of unsteamed and steamed beech wood with water steam. Polymers.

[45] Dudiak M., Dzurenda L. (2023). The effect of sunlight on the color change of steamed birch wood. Acta Fac. Xylologiae Zvolen.

[46] Kúdela J., Sikora A., Gondáš L. (2023). Wood surface finishing with transparent lacquers intended for indoor use, and the colour resistance of these surfaces during accelerated aging. Polymers.

[47] Pavlič M., Petrič M., Žigon J. (2021). Interactions of coating and wood flooring surface system properties. Coatings.

[48] Salca E.A., Krystofiak T., Lis B., Hiziroglu S. (2021). Glossiness evaluation of coated wood surfaces as function of varnish type and exposure to different conditions. Coatings.

